# Calibration-Free Gait Assessment by Foot-Worn Inertial Sensors

**DOI:** 10.3389/fdgth.2021.736418

**Published:** 2021-11-04

**Authors:** Daniel Laidig, Andreas J. Jocham, Bernhard Guggenberger, Klemens Adamer, Michael Fischer, Thomas Seel

**Affiliations:** ^1^Control Systems Group, Technische Universität Berlin, Berlin, Germany; ^2^Institute of Physiotherapy, FH JOANNEUM University of Applied Sciences, Graz, Austria; ^3^Vamed Rehabilitation Center Kitzbuehel, Kitzbuehel, Austria; ^4^Ludwig Boltzmann Institute for Rehabilitation Research, Vienna, Austria; ^5^Hannover Medical School MHH, Clinic for Rehabilitation Medicine, Hannover, Germany; ^6^Department Artificial Intelligence in Biomedical Engineering, Friedrich-Alexander-Universität Erlangen-Nürnberg, Erlangen, Germany

**Keywords:** inertial sensors, IMU, human motion analysis, gait analysis, gait assessment, gait phases, rehabilitation, walking

## Abstract

Walking is a central activity of daily life, and there is an increasing demand for objective measurement-based gait assessment. In contrast to stationary systems, wearable inertial measurement units (IMUs) have the potential to enable non-restrictive and accurate gait assessment in daily life. We propose a set of algorithms that uses the measurements of two foot-worn IMUs to determine major spatiotemporal gait parameters that are essential for clinical gait assessment: durations of five gait phases for each side as well as stride length, walking speed, and cadence. Compared to many existing methods, the proposed algorithms neither require magnetometers nor a precise mounting of the sensor or dedicated calibration movements. They are therefore suitable for unsupervised use by non-experts in indoor as well as outdoor environments. While previously proposed methods are rarely validated in pathological gait, we evaluate the accuracy of the proposed algorithms on a very broad dataset consisting of 215 trials and three different subject groups walking on a treadmill: healthy subjects (*n* = 39), walking at three different speeds, as well as orthopedic (*n* = 62) and neurological (*n* = 36) patients, walking at a self-selected speed. The results show a very strong correlation of all gait parameters (Pearson's *r* between 0.83 and 0.99, *p* < 0.01) between the IMU system and the reference system. The mean absolute difference (MAD) is 1.4 % for the gait phase durations, 1.7 cm for the stride length, 0.04 km/h for the walking speed, and 0.7 steps/min for the cadence. We show that the proposed methods achieve high accuracy not only for a large range of walking speeds but also in pathological gait as it occurs in orthopedic and neurological diseases. In contrast to all previous research, we present calibration-free methods for the estimation of gait phases and spatiotemporal parameters and validate them in a large number of patients with different pathologies. The proposed methods lay the foundation for ubiquitous unsupervised gait assessment in daily-life environments.

## 1. Introduction

Walking is a central activity of daily life, and restrictions of this ability lead to a reduction in the quality of life (1, 2). Therefore, gait analysis is an important tool in different medical and therapeutic fields (3, 4). The measurement of various gait characteristics can either facilitate diagnosis or be used to track the progress of rehabilitation. Gait can be measured by spatial (e.g., step or stride length) and temporal (e.g., stride time, cadence) parameters, relative durations of gait phases, and kinematic and kinetic gait variables (5). These parameters are used to quantify gait deviation in both clinical practice and research, and their use varies with the medical field, the research question, and the analysis options. While gait assessment in clinical practice is mostly based on visual observation by medical experts (6), it is desirable to support expert knowledge and time by objective measurements. This is also important because relevant gait changes are often too subtle to be detected by the naked eye (7).

Traditionally, sensor-based gait assessment is performed with stationary systems like marker-based optical motion tracking, instrumented treadmills, or pressure-sensitive walkways (6, 8). Besides being expensive, one major drawback of those systems is that they are limited to a small capture space or require the subject to walk on a treadmill (4, 9–12). Furthermore, the use of walking aids is often not possible or restricted in combination with such systems.

A promising, more ambulatory, and less restrictive alternative is inertial gait analysis, i.e., gait analysis with inertial sensor technology. Lightweight and battery-powered inertial measurement units (IMUs) are used, which transmit the data wirelessly.

**Figure 1:**
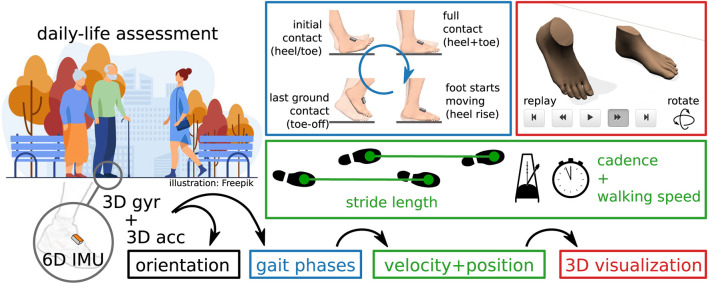
Inertial gait analysis can be realized with two miniaturized IMUs on the shoes, enabling daily-life assessment outside of laboratory environments. From the raw sensor data, orientation, gait phases, and velocity and position trajectories can be estimated. Parameters commonly used in gait analysis, such as stride length, cadence, and walking speed, can easily be derived from this.

The transition from expensive stationary systems to small wearable sensors opens up possibilities that go beyond replacing the measurement technology used for gait assessment in a clinical setting. Integrating objective long-term gait monitoring in day-to-day life—as illustrated in Figure 1—could provide more powerful tools for clinicians to help patients in rehabilitation but also to gain further insights into disease progression. Furthermore, non-obtrusive wearable plug-and-play systems facilitate applications in neuroprosthetics (13) or exoskeletons and can be used to provide biofeedback (14). In the last years, wireless battery-powered IMUs have become smaller, lighter, more accurate, and at the same time cheaper and more energy-efficient, and it is to be expected that this development is going to continue. For those new trends, it is important to develop methods that can provide a wide variety of gait parameters that are useful to medical experts. At the same time, the methods need to be robust so that the system can be used by patients in unsupervised settings, outdoors as well as indoors.

It has been shown by previous contributions (15–17) that major gait parameters can be determined with two IMUs that are placed on the feet or the shoes, as illustrated in Figure 1. This includes stride length, gait phase durations (e.g., stance and swing percentage), and also the cadence and walking speed.

Our aim is to propose methods for gait assessment that meet the requirements for day-to-day life monitoring in unsupervised settings and that are validated on a broad group of subjects including patients with various gait pathologies. The proposed methods do not assume any fixed orientation of the sensor with respect to the foot and do not require the subject to perform dedicated calibration movements. Furthermore, magnetometers are not used since the magnetic field is known to be severely disturbed in indoor environments (18). This makes the use of inertial gait analysis easy and practical in clinical settings and facilitates future applications of ubiquitous gait analysis in home environments.

The remainder of the article is structured as follows. In section 2, we briefly review existing methods for IMU-based spatiotemporal gait parameter estimation. In section 3, we describe the proposed methods, which we then validate in section 4 using experimental data of 98 orthopedic and neurological patients, as well as 39 healthy subjects walking at different speeds. The results are discussed in section 5, and section 6 provides conclusions.

## 2. Brief Review of IMU-Based Spatiotemporal Gait Parameter Estimation

Several methods have been proposed that employ inertial sensors to obtain spatiotemporal gait parameters. In the following, we present a brief overview of the current state of the art and summarize the different hardware setups that are used, which parameters are calculated, and how the methods were validated. Table 1 categorizes 23 publications that provide a range of examples for the variety of existing approaches in the estimation of spatiotemporal gait parameters with inertial sensors.

**Table 1:** Overview of IMU-based spatiotemporal gait parameter estimation literature.

Employed sensor setup	
2 IMUs on feet/shoes	(15, 17, 19–31)
2 IMUs on shank	(13, 16, 27, 30, 32, 33)
3 or more IMUs	(34–37)
Detected gait phases	
Stance/swing	(13, 16, 17, 22, 23, 27, 30, 32, 33, 35, 37)
4 unilateral events	(15, 19–21, 25, 26)
Single/double support	(29, 36)
Ground truth used for evaluation	
Optical motion capture	(20, 25, 26, 28, 29, 31, 34, 37)
Pressure-sensitive walkways	(16, 17, 23, 32, 33, 35)
Instrumented treadmills	(24, 27)
Pressure insoles	(15, 30)
Others/none	(19, 21, 22, 36)
Non-healthy subjects included in evaluation	
None (healthy only)	(21, 22, 24–28, 34, 36, 37)
≤ 20	(20, 30, 31, 33, 35)
>20	(15–17, 23, 29, 32)

*A total of 23 publications that describe estimation of spatiotemporal gait parameters with IMUs are categorized based on sensor setup, detected gait phases, and the ground truth and number of non-healthy subjects for evaluation*.

There are different hardware setups, based on the number of inertial sensors and their placement. The chosen setup has an impact on which and how many parameters can be derived from the measured data. The most commonly used setup consists of two IMUs. As shown in Table 1, sensors are typically placed on the feet or shoes and sometimes on the shank. This setup is occasionally extended by adding a third sensor on the pelvis or lumbar spine (34, 35). Note that it has even been shown that temporal gait events can be obtained from a single IMU at the pelvis (38), but the potential for extracting further spatial parameters is limited. Full (lower) body motion tracking opens up additional possibilities, as demonstrated with 7 IMUs on the lower body and pelvis in (37) and with 8–15 IMUs in (36). Another, less common, option consists of combining inertial sensors with further measurement devices, e.g., a camera on one foot and LEDs on the other foot to facilitate the direct measurement of relative positions (39).

Some methods require that a known orientation of the sensor axes with respect to the anatomical foot axes has to be ensured by precise placement. Many methods in the literature are based on such assumptions, including (13, 15–17, 22, 23, 25, 26, 30–33). In practice, however, ensuring a precise placement is a challenge, especially in non-supervised application scenarios and during activities of daily life. Alternatives are to develop methods that are agnostic to the sensor-to-segment orientation—e.g., by only relying on signal norms—or to determine this orientation in a process commonly called anatomical calibration (40). For setups with more sensors, there are recently developed methods that facilitate automatic anatomical calibration by exploiting kinematic constraints of the respective joints without requiring the subject to perform precise calibration movements (41, 42). For those setups, the linking of the sensors to the body segments poses another challenge to a plug-and-play approach, which can be solved by automatic pairing methods (43).

The calculation of spatiotemporal gait parameters is usually implemented in a two-stage approach. In a first step, gait events and corresponding gait phases are detected. In a second step, spatial parameters are calculated.

Existing methods vary in the set of gait events or phases that are detected. In many cases, the focus is only on the separation between stance and swing (cf. Table 1), although sometimes additional events, such as mid-swing (33), are also detected. It is also common to detect four events that occur during the gait cycle and are only defined by the ipsilateral (same) foot. Those events are initial contact, full contact, heel rise, and toe-off, although the terminology varies. Despite being common practice in gait analysis (5, 44), employing bilateral information, i.e., combining information from both feet to define the gait phase, is far less common in IMU-based gait analysis. One example is (36) in which single and double limb support durations are calculated.

There are various approaches for detection of gait events using inertial sensors. It has been shown that exploiting features of the angular rate signal in the sagittal plane is sufficient to achieve reliable gait event detection (16, 22, 25, 30, 32). Many other methods use both accelerometers and gyroscopes and detect characteristic signal features in the inertial sensor data, including (13, 15, 17, 19–21, 23, 31, 33). Sometimes automatic adaption mechanisms are used to adjust thresholds based on the subject's walking style (19–21, 33). An alternative to the signal-based methods is to rely on a kinematic model to detect gait events (36, 37). Machine learning methods, often based on hidden Markov models (26, 35), are also used for event detection [cf. (45)].

In addition to the detection of gait events, spatial parameters such as stride length and walking speed are often calculated. Those parameters are obtained by either signal integration, human gait models, or by machine learning methods (46). By far the most common approach is numerical strapdown integration of the accelerations (16, 17, 22, 27, 28, 31, 32, 37). The cyclic nature of gait and the fact that there is frequent ground contact are exploited to correct for drift that is due to double integration. It has been shown that Fourier-based integration is an alternative to numerical integration (34), that spatial parameters can be obtained from kinematic models (27, 36), and that convolutional neural networks can also be used to estimate spatial parameters (23).

Most publications focus on common spatiotemporal parameters such as stride length, walking speed, and cadence. Other than those spatiotemporal parameters, there is a multitude of spatiotemporal gait parameters that are relevant in a clinical context for various pathologies (6). Examples that can be estimated using inertial sensors include step width (37), swing width (31, 37), incline (22), and foot clearance (47).

Some publications (13, 19–21, 25, 33) focus on real-time detection of events, e.g., to trigger functional electrical stimulation (FES). While the approaches used are usually similar to the ones used in offline gait analysis, this typically implies a focus on minimizing the detection delay rather than the accuracy of the reported values.

As shown in Table 1, evaluation is often performed with marker-based optical motion capture as ground truth. Systems based on the detection of pressure, such as pressure-sensitive walkways, instrumented treadmills, and pressure insoles, are a common alternative. In some cases, no validation with respect to a gold standard is performed. Instead, the settings of a (calibrated) treadmill are used for walking speed and incline (22), a manually counted number of steps is combined with the detection of irregularities (21), validation is performed by visual inspection of the results (19), or the focus is only on test-retest reliability (36).

Even though it has been shown that the accuracy of gait analysis methods decreases when applied to non-healthy subjects (45), the evaluation of inertial gait analysis methods is often only based on healthy subjects. When data obtained from non-healthy subjects is part of the evaluation, the number of subjects is often small, for example five transfemoral amputees (20), 10 stroke patients (33), 10 hemiparetic patients and 10 Huntington's disease patients (35), or 10 patients with Parkinson's disease (31).

To the best of our knowledge, few publications (15–17, 23, 29, 32) exist which propose methods for IMU-based spatiotemporal gait parameter estimation *and* validate the methods on a larger set of subjects with gait pathologies. In the following, we briefly summarize those publications.

In (15), sensors are placed on the forefoot in a known orientation, and four different unilateral gait events are detected based on features of the angular velocity in the sagittal plane, the norm of the accelerometer signal, and the derivative of angular velocity norm. Using pressure insoles as reference, the method is validated on 10 healthy and 32 orthopedic subjects.

The commercial Gait Up system is evaluated in (29) with 25 subacute stroke patients as subjects and marker-based optical motion capture as reference.

Gait events and stride length are calculated in (16) based on shank-mounted IMUs. Events are detected based on the angular rate in the sagittal plane, and stride length is obtained via double integration of the accelerations. The latter relies on the proprietary orientation estimation algorithm provided by the sensor manufacturer. Experimental evaluation is performed using the GAITRite pressure-sensitive walkway as reference on 10 healthy elderly and 30 non-healthy subjects.

In (32), the same method is validated on a much larger group of subjects, consisting of 236 community-living older adults, including 31 mild cognitive impaired subjects and 125 Parkinson's disease patients.

In (17), IMUs are placed laterally on the shoe in a fixed orientation, stance, and swing durations are calculated based on characteristic signal features, and the stride length is obtained via double integration. The method is evaluated using a large data set of 101 geriatric inpatients, with reference data obtained from a GAITRite pressure-sensitive walkway.

Using the same gait event detection method and the same data set for evaluation as (17), Hannink et al. (23) estimates stride length, stride width, mediolateral change in foot angle, heel contact times, and toe contact times using deep convolutional neural networks.

In summary, the main shortcoming of existing approaches for the vision of plug-and-play ambulatory gait analysis is that most methods—especially those with broad validation—require a precise attachment of the sensor to the subject's foot. Some methods only focus on gait events and do not provide spatial parameters, and some methods rely on proprietary algorithms of the sensor manufacturers. Furthermore, very few of the proposed methods are validated on a large group of subjects with diverse gait pathologies.

In the following section, we propose a set of methods that combine the valuable achievements of existing methods with additional features that overcome the remaining limitations.

## 3. Methods

In the following, we propose a set of methods to determine gait parameters from two IMUs attached to the foot. The proposed methods are based on the following assumptions and requirements: An IMU is attached to each foot (or shoe) in an arbitrary orientation. This implies that the proposed method does not make any assumption about the orientation of the sensor coordinate system, which means it does not require any specific sensor axis to be aligned with an anatomical or functional axis of the foot. In order to avoid artifacts caused by toe or ankle motions, and also to not limit the subject's freedom of movement, we propose to attach the IMU on the instep, i.e., the dorsal side of the midfoot. We obtain the accelerometer and gyroscope readings of both IMUs at a fixed sampling rate (typically in the range 50–1,000 Hz). We assume that data for several steps is processed at once, which allows us to employ non-causal signal processing to increase the accuracy compared to sample-by-sample real-time capable methods. This processing can either be performed in batches while the subject is walking, e.g., for use in biofeedback applications, or after the recording is completed. During the recording, the subject walks either on a treadmill or an indoor or outdoor ground.

**Figure 2:**
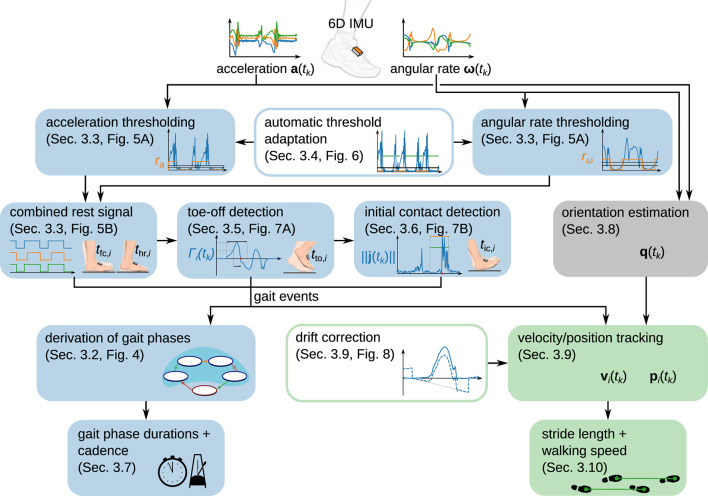
Overview of the proposed modular set of methods to determine spatiotemporal gait parameters from foot-worn IMUs. While gait phase durations and cadence are determined from gait events, stride length and walking speed are derived from a position trajectory obtained via piecewise strapdown integration of the acceleration.

The set of methods that we propose is explained in the following subsections, and the presentation is structured as follows. Separately for each foot, we use the recorded sensor data to separate phases in which the foot is in full contact with the ground from phases in which the foot moves, i.e., we detect when strides take place (section 3.3). For each detected stride, we then detect toe-off (section 3.5) and initial contact (section 3.6). The gait events from the ipsilateral and contralateral foot are combined to define gait phases. We calculate the relative duration of each gait phase and the cadence (section 3.7). We then estimate the sensor orientation by sensor fusion of the gyroscope and accelerometer readings (section 3.8) and double-integrate the acceleration to obtain a position trajectory (section 3.9). From this position trajectory, we obtain the stride length and the walking speed (section 3.10). Figure 2 provides an overview of the proposed set of methods.

In the remainder of this section, we define parameters used by the method. For an overview of those parameters and proposed values, please refer to Table 2 in section 4. Our aim is to define the parameters in a way that they are not sensitive to different gait styles or velocities. In section 4, we demonstrate that this approach works by only employing one common set of parameters for validation on a very broad data set with healthy and non-healthy subjects walking at different speeds.

**Table 2:** Parameter values used for the proposed IMU-based methods.

**Symbol**	**Description**	**Value**
*h*_*a*_	Hysteresis factor for acceleration	0.23
*h*_ω_	Hysteresis factor for angular rate	0.23
*w*_*a*_	Factor for *a*_th_ auto-tuning	0.85
*a*_th,min_	Lower bound for *a*_th_	1.8 m/s^2^
*w*_ω_	Factor for ω_th_ auto-tuning	0.8
ω_th,min_	Lower bound for ω_th_	0 rad/s
*T*_0,min_	Minimum duration of zero-phase	120 ms
*T*_1,min_	Minimum duration of one-phase	180 ms
*j*_win_	Ratio of the window to look for initial contact	0.7
*j*_th_	Threshold for jerk norm (relative to maximum)	0.95
*T*_*a*_	Time constant for acceleration moving average filter	8.0 s

*This parametrization is used for the processing of all trials, regardless of gait pathology, walking speed, or style, in order to show that the method works well without tuning the parameters for specific gait characteristics*.

### 3.1. Notation

Denote the accelerometer readings $\text{a}\left(t_{k}\right)\in {\mathbb{R}}^{3}$ and the gyroscope readings $\omega \left(t_{k}\right)\in {\mathbb{R}}^{3}$, sampled at times *t*_*k*_ = *kT*_s_, *k* ∈ {1 .. *N*}, *T*_s_ ∈ ℝ_>0_.

In the following, all times *t* with any index are multiples of *T*_*s*_. If any calculation yields a time that is not a multiple of *T*_*s*_, we assume that this value is rounded to the nearest multiple of *T*_*s*_ and do not explicitly write this for the sake of a compact notation. Furthermore, any summation over τ should be interpreted as a summation with a non-integer step size of *T*_*s*_, i.e., we simply write $\overset{t_{2}}{\underset{\tau =t_{1}}{\sum }}x\left(\tau \right)$ instead of the longer but mathematically precise notation $\overset{k_{2}}{\underset{k=k_{1}}{\sum }}x\left(t_{k}\right),k_{1}=\frac{t_{1}}{T_{\text{s}}},k_{2}=\frac{t_{2}}{T_{\text{s}}}$.

Unit quaternions in vector notation are used to represent rotations and orientations (48). When a quaternion is used to represent the sensor orientation, it is the rotation from an inertial reference frame with the *z*-axis pointing up (and arbitrary heading) to the coordinate system of the sensor. In the context of quaternion multiplication, which we denote by ⊗, three-dimensional vectors are implicitly regarded as quaternions with zero real part.

Furthermore, ***v***^⊺^ denotes the transpose of the vector ***v***.

### 3.2. Gait Events and Gait Phases

**Figure 3:**
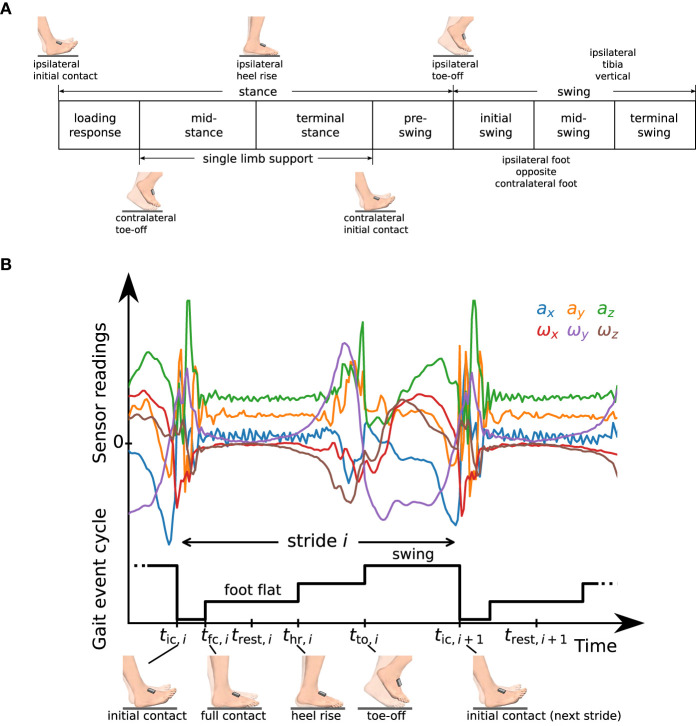
**(A)** Definition of gait phases as used in standard literature [cf. (44)], and transitions based on gait events of the ipsilateral and contralateral foot. **(B)** Raw accelerometer and gyroscope sensor readings and representation of the gait event cycle with a staircase-shaped signal. We define time instants *t*_ic,*i*_, *t*_fc,*i*_, *t*_hr,*i*_, *t*_to,*i*_ that mark characteristic events and a rest instant *t*_rest,*i*_ in the middle of the phase in which the foot is fully on the ground (foot flat).

According to standard literature (44) and as illustrated in Figure 3A, the gait cycle starts at initial contact. Each stride can be separated into *stance* and *swing*. Stance consists of the gait phases *loading response, mid-stance, terminal stance*, and *pre-swing*. Swing can be separated into *initial swing, mid-swing*, and *terminal swing*. The combination of mid-stance and terminal stance is called *single limb support* and corresponds to the swing phase of the contralateral foot. In standard literature (44), the initial contact is commonly considered to be a very short gait phase with a duration of 2 %. As it is common practice in IMU-based gait analysis (15, 19, 21, 26), we define the initial contact as an event without duration. Note that sometimes the initial contact is also called foot strike (26) or heel strike (15).

The separation between stance and swing and the separation of stance into loading response, mid-stance, terminal stance, and pre-swing is defined based on three events that describe a change of ground contact of the feet: *initial contact, heel rise*, and *toe-off*. In contrast, the separation of swing into initial swing, mid-swing, and terminal swing is based on positional information of the feet and on the tibia orientation. The gait phases are defined based on bilateral events, i.e., the gait phase of the ipsilateral foot is not only described based on the events of the same (ipsilateral) foot but also based on toe-off and initial contact of the other (contralateral) foot.

We will now describe how we determine five of those gait phases (swing and the four sub-phases of stance) using IMUs in a two-step approach. First, we detect four gait events independently for each foot. We then use this gait event cycle of both feet to derive gait phases for each foot.

To this end, for each stride *i* ∈ {1 .. *M*}, we define the following events that we want to detect independently for the right and left foot from the raw measurement data of the corresponding IMU:

- initial contact – *t*_ic,*i*_
- full contact – *t*_fc,*i*_
- heel rise – *t*_hr,*i*_
- toe-off – *t*_to,*i*_.

Note that in addition to the three events used to define gait phase transitions in Figure 3A, we introduce an event called *full contact* that indicates that the foot is in full contact with the ground. For various processing steps, such as zero-velocity updates and position integration, we further define a rest instant *t*_rest,*i*_ at the middle of the foot flat phase, i.e.,


(1) $$\begin{array}{l} t_{\text{rest},i}≔\frac{1}{2}\left(t_{\text{fc},i}+t_{\text{hr},i}\right). \end{array}$$

See Figure 3B for a plot of the raw accelerometer and gyroscope data measured during one stride along with a graphical representation of the gait event cycle defined by the introduced events. In the following subsections, we will describe in detail how we determine those time instants from the raw sensor data.

**Figure 4:**
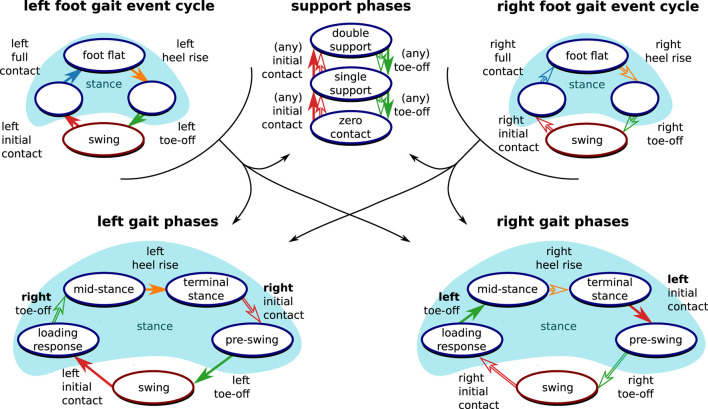
Derivation of clinically relevant gait phases from the gait event cycles. Following standard literature (44), events from both the left foot (filled arrows) and the right root (outlined arrows) are necessary to define the gait phase of each foot. Furthermore, support phases based on the number of feet that are in contact with the ground can be defined based on the gait events.

After having determined the gait events for both feet, we use the gait event cycles from both feet to determine the gait phase according to the commonly used definitions by (44). As shown in Figure 4, finite automata for the gait phases of the left and right foot are each driven by the gait event cycles of both feet.

Since time instances from both sensors are used for the definition of the gait phase transitions, both feet must be equipped with sensors, and precise time synchronization is required. However, note that the separation into stance and swing directly follows from the gait event cycle (as shown in Figure 4) and is independent of the contralateral foot. Therefore, we can determine stance and swing regardless of the synchronization between the sensors. This is also useful if only one foot is equipped with a sensor and facilitates on-chip data processing.

Note that the three sub-phases of stance in the gait event cycle hold further information that is not directly captured by the standard gait phase definitions as given in Figure 3A. We denote the phase from *t*_fc,*i*_ to *t*_hr,*i*_, in which the foot is fully on the ground, as *foot flat*. Note that the other two sub-phases of the stance phase, *t*_ic,*i*_ to *t*_fc,*i*_ and *t*_hr,*i*_ to *t*_to,*i*_, are sometimes called loading response and pre-swing (19, 21) but do not correspond to the phases with the same name as defined in standard literature (44).

Furthermore, as also shown in Figure 4, time-synchronized events from both feet also allow for the distinction of *double support, single support*, and *zero-contact* phases, which occur only during running (44).

### 3.3. Foot Flat Detection

As the first step of gait phase detection, the phases in which the foot is fully on the ground (foot flat) are detected. When the foot is fully on the ground, the Euclidean norm of the accelerometer readings will be close to 9.81 m/s^2^, and the norm of the gyroscope readings will be close to zero. During a stride, we typically will see an increase in the signal norms. However, it is possible that during the motion phase there are long periods with only small changes of velocity or small rotations. To obtain a robust stride detection, we, therefore, first find activity using either the accelerometer or the gyroscope readings and then combine this information.

For an acceleration-based rest signal *r*_*a*_(*t*_*k*_), we consider the absolute difference of the norm from 9.81 m/s^2^,


(2) $$\begin{array}{l} a\left(t_{k}\right)≔|∥a\left(t_{k}\right)∥-9.81|, \end{array}$$

and perform acausal thresholding using a threshold *a*_th_ and a hysteresis factor *h*_*a*_ by applying hysteresis in forward and backward direction, i.e.,


(3) $$r_{a}^{*}(t_{k})≔\{\begin{array}{ll} 1 & a(t_{k})>(1+h_{a})a_{\text{th}}\\ 0 & a(t_{k})<(1-h_{a})a_{\text{th}}\\ r_{a}(t_{k-1}) & \text{otherwise} \end{array}$$

(4) $$r_{a}(t_{k})≔\{\begin{array}{ll} 1 & r_{a}^{*}(t_{k})=1\\ 0 & a(t_{k})<(1-h_{a})a_{\text{th}}\\ r_{a}(t_{k+1}) & \text{otherwise} \end{array}$$

with $r_{a}^{*}\left(0\right)=0$ and $r_{a}\left(t_{N}\right)=r_{a}^{*}\left(t_{N}\right)$. In the resulting signal, zero-phases shorter than *T*_0,min_ are set to one, and afterward, one-phases shorter than *T*_1,min_ are set to zero.

**Figure 5:**
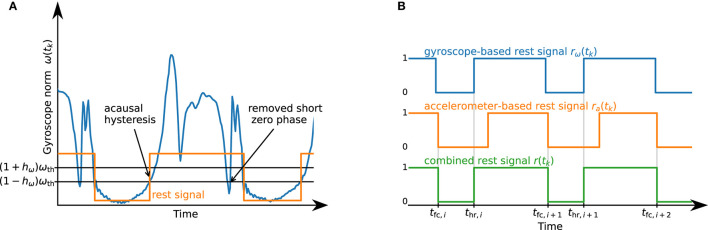
**(A)** Illustration of the thresholding algorithm. Acausal hysteresis and the removal of short phases ensure the robust detection of the desired rest phase. **(B)** Illustration of the combination of *r*_ω_(*t*_*k*_) and *r*_*a*_(*t*_*k*_) into *r*(*t*_*k*_). By using the *OR* combination of the accelerometer- and gyroscope-based signals, we are able to robustly detect when the foot is not fully on the ground.

The same acausal thresholding with the removal of short phases is applied to the gyroscope norm signal ω(*t*_*k*_): = ∥**ω**(*t*_*k*_)∥ using a threshold ω_th_ and hysteresis factor *h*_ω_, which yields a gyroscope-based rest signal *r*_ω_(*t*_*k*_). See Figure 5A for an illustration of the thresholding method.

Both rest signals, *r*_*a*_(*t*_*k*_) and *r*_ω_(*t*_*k*_), are combined into *r*(*t*_*k*_), which is set to one if at least one of the two signals is one. Afterward, zero-phases shorter than *T*_0,min_ are set to one, and then one-phases shorter than 2*T*_1,min_ are set to zero. This process is illustrated in Figure 5B. Each zero-to-one transition of the resulting signal marks a heel rise *t*_hr,*i*_, and each one-to-zero transition marks a full contact *t*_fc,*i*+1_.

### 3.4. Automatic Threshold Adaptation

A common issue with thresholding approaches is that the thresholds have to be adapted based on gait velocity and also other gait and sensor characteristics (19, 20). Therefore, instead of performing the thresholding of the accelerometer and gyroscope norm using manually tuned thresholds *a*_th_ and ω_th_, we propose an algorithm that automatically determines these thresholds for each trial based on the measured data.

The threshold *a*_th_ is determined using an iterative algorithm similar to (49), with *l* being the iteration index and *w*_*a*_ being a weighting parameter:


(5) $$\begin{array}{l} a_{\text{th},0}=\frac{1}{2}\left(\underset{t_{k}\in \left[t_{1},t_{N}\right]}{\max}a\left(t_{k}\right)+\underset{t_{k}\in \left[t_{1},t_{N}\right]}{\min}a\left(t_{k}\right)\right) \end{array}$$

(6) $$\begin{array}{l} T^{+}=\left\{t_{k}\in \left[t_{1},t_{N}\right]|a\left(t_{k}\right)>a_{\text{th},l}\right\} \end{array}$$

(7) $$\begin{array}{l} T^{-}=\left\{t_{k}\in \left[t_{1},t_{N}\right]|a\left(t_{k}\right)\leq a_{\text{th},l}\right\} \end{array}$$

(8) $$\begin{array}{l} a_{\text{th},l+1}=\frac{w_{a}}{\left|T^{-}\right|}\underset{t_{k}\in T^{-}}{\sum }a\left(t_{k}\right)+\frac{1-w_{a}}{\left|T^{+}\right|}\underset{t_{k}\in T^{+}}{\sum }a\left(t_{k}\right). \end{array}$$

**Figure 6:**
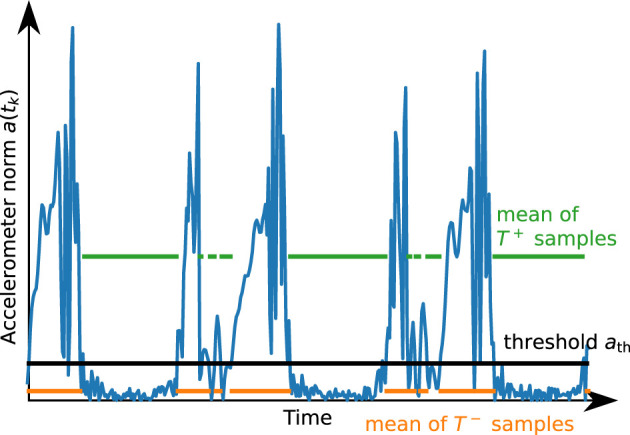
Illustration of the result of the automatic thresholding algorithm for a short segment of accelerometer data. The threshold *a*_th_ is chosen such that the mean of the values above and the mean of the values below are in a certain proportion.

We perform 200 iterations to ensure convergence, i.e., *a*_th_ := *a*_th,200_. Figure 6 illustrates the result of this process. Further, we define a lower bound *a*_th,min_ for this threshold.

Similarly, we determine the threshold ω_th_ based on the gyroscope norm ω(*t*_*k*_) and a weighting factor *w*_ω_.

### 3.5. Toe-Off Detection

After determining heel rise and full contact, we want to detect the beginning of the swing phase, i.e., the toe-off. During toe-off, the foot first rotates approximately along the mediolateral axis as the heel rises, then loses contact with the ground and rotates in the opposite direction. An inertial sensor attached to the foot cannot directly measure when the foot fully loses contact with the ground, in contrast to, e.g., pressure-sensitive walkways. Note that the accuracy of toe-off detection using pressure sensors also depends on calibration and the chosen thresholds (12).

As rotation can be measured precisely with IMUs, we exploit the fact that the direction of rotation of the foot changes when transitioning from the phase in which the heel rises while the toe stays on the ground to the phase in which the toe leaves the ground. This approach is commonly used in existing literature, as detailed in section 2. However, most methods directly rely on the angular rate measured in the sagittal plane and thereby require at least one sensor axis to be well-aligned with a functional axis of the foot.

To be independent of the sensor orientation and also to obtain a reliable detection if the subject exhibits strong inversion or eversion during toe-off, we define a signal called tilt-rate Γ_*i*_(*t*_*k*_), from each heel rise *t*_hr,*i*_ to the subsequent full contact *t*_fc,*i*+1_, as


(9) $$\begin{array}{l} \Gamma_{i}\left(t_{k}\right)≔\omega {\left(t_{k}\right)}^{⊺}\frac{\sum_{\tau =t_{\text{hr},i}}^{t_{k}}\omega \left(\tau \right)}{\sum_{\tau =t_{\text{hr},i}}^{t_{k}}\omega \left(\tau \right)},t_{k}\in \left[t_{\text{hr},i},t_{\text{fc},i+1}\right]. \end{array}$$

The rationale behind the definition of the tilt-rate Γ_*i*_(*t*_*k*_) is to identify the main axis of rotation since the last heel rise and compute the current rate of rotation around this main axis. This enables us to detect a zero-crossing of the main rotation without making any assumptions on the orientation of the sensor with respect to the foot.

**Figure 7:**
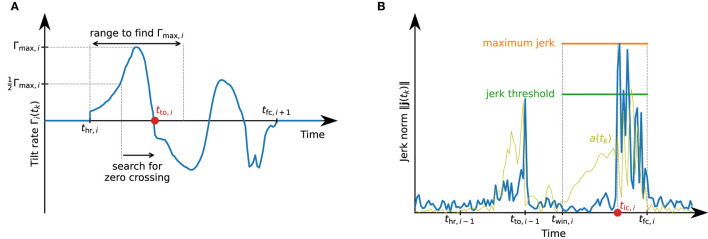
Detection of toe-off and initial contact events that define the swing phase. **(A)** Illustration of the toe-off detection. Between heel rise and full contact, the tilt rate might exhibit multiple local maxima and zero-crossings. For a robust detection of the correct zero-crossing, we first find the maximum value during the first half of the phase from *t*_hr,*i*_ to *t*_fc,*i*+1_ and search for the first zero-crossing after the tilt rate has reached half of this maximum. **(B)** Illustration of the initial contact detection based on the jerk norm. Note how the jerk norm reflects the sudden change when the foot touches the ground much better than the accelerometer norm signal *a*(*t*_*k*_).

In general, the tilt-rate Γ_*i*_(*t*_*k*_) will exhibit a change of sign after a distinct peak (cf. Figure 7A). As there might be noise, leading to frequent sign changes right after *t*_hr,*i*_, as well as large peaks later during the stride, we propose the following strategy to robustly determine the sign change of interest:

During the first half of the movement phase, let Γ_max,*i*_ denote the maximum value of Γ_*i*_(*t*_*k*_), i.e.,


(10) $$\begin{array}{l} \Gamma_{\text{max},i}≔\underset{t_{k}\in \left[t_{\text{hr},i},\frac{1}{2}\left(t_{\text{hr},i}+t_{\text{fc},i+1}\right)\right]}{\max}\Gamma_{i}\left(t_{k}\right). \end{array}$$

We then find the first time instant for which $\Gamma_{i}\left(t_{k}\right)\geq \frac{1}{2}\Gamma_{\text{max},i}$. Starting from this time instant, we find the first time instant at which Γ_*i*_(*t*_*k*_) ≤ 0. We assume this time instant to be the toe-off *t*_to,*i*_, i.e., the start of the swing phase. Figure 7A illustrates this process.

Note that *t*_to,*i*_ is defined based on a feature of the rotation of the foot and not directly as the lift-off of the toes. Using the maximum of the tilt rate (or any weighted average of the maximum and zero-crossing time instant) are also plausible approaches.

### 3.6. Initial Contact Detection

The initial contact marks the beginning of the loading response and can be detected by the jerk, i.e., the change of acceleration, caused by the foot touching the ground. We calculate the jerk using the first-order backward difference approximation, i.e.,


(11) $$\begin{array}{l} j\left(t_{k}\right)≔\frac{1}{T_{\text{s}}}\left(a\left(t_{k}\right)-a\left(t_{k-1}\right)\right). \end{array}$$

For every stride, we only consider a sub-window of the phase between toe-off and the beginning of the subsequent foot-flat phase and denote the start time of this window as *t*_win,*i*_ : = *j*_win_*t*_to,*i*−1_ + (1 − *j*_win_)*t*_fc,*i*_,*j*_win_ ∈ [0, 1]. In this time window, we first determine the maximum value of the jerk norm, i.e.,


(12) $$\begin{array}{l} j_{\text{max},i}≔\underset{t_{k}\in \left[t_{\text{win},i},t_{\text{fc},i}\right]}{\max}∥j\left(t_{k}\right)∥. \end{array}$$

We then mark the first time instant in this window with ∥***j***(*t*_*k*_)∥ ≥ *j*_th_*j*_max,*i*_ as the start of the loading response *t*_ic,*i*_. See Figure 7B for an illustration of the initial contact detection.

### 3.7. Stride and Gait Phase Durations and Cadence

For each detected stride, we calculate the stride duration as the duration from one initial contact to the subsequent initial contact of the same foot, i.e.,


(13) $$\begin{array}{l} T_{\text{stride},i}≔t_{\text{ic},i+1}-t_{\text{ic},i}. \end{array}$$

For each detected stride, the duration of the swing phase is the time between toe-off and initial contact of the subsequent stride, i.e.,


(14) $$\begin{array}{l} T_{\text{swing},i}≔t_{\text{ic},i+1}-t_{\text{to},i}. \end{array}$$

The stance duration is the remaining duration of the stride:


(15) $$\begin{array}{l} T_{\text{stance},i}≔T_{\text{stride},i}-T_{\text{swing},i}. \end{array}$$

Since relative gait phase durations are easier to interpret, we calculate


(16) $$\begin{array}{ll} T_{\text{swing,rel},i} & ≔\frac{T_{\text{swing},i}}{T_{\text{stride},i}}, \end{array}$$

(17) $$\begin{array}{ll} T_{\text{stance,rel},i} & ≔\frac{T_{\text{stance},i}}{T_{\text{stride},i}}. \end{array}$$

Similarly, for every stride we calculate relative gait phase durations for loading response *T*_lr,rel,*i*_, single limb support *T*_sl,rel,*i*_, terminal stance *T*_ts,rel,*i*_, and pre-swing *T*_ps,rel,*i*_, based on the bilateral gait phases as defined in Figure 3A. Note that, analogously, we can also calculate absolute and relative durations for all other gait phases defined in Figure 3A.

To calculate the cadence, we multiply the inverse of the stride duration by two in order to express the cadence as the number of steps per minute instead of strides per minute, i.e.,


(18) $$\begin{array}{l} c_{i}≔\frac{2}{T_{\text{stride},i}}. \end{array}$$

### 3.8. Orientation Estimation

By fusing the gyroscope and accelerometer measurements, we obtain an estimate of the sensor orientation with respect to a global frame that has a vertical *z*-axis and an arbitrary heading.

Starting with an arbitrary initial orientation **q**_ω_(0), e.g., ${\left[\begin{array}{cccc} 1 & 0 & 0 & 0 \end{array}\right]}^{\text{T}}$, we perform gyroscope strapdown integration


(19) $$\begin{array}{l} {\text{q}}_{\omega }\left(t_{k}\right)≔{\text{q}}_{\omega }\left(t_{k-1}\right)⊗{\left[\begin{array}{cc} \cos\left(\frac{T_{\text{s}}}{2}∥\omega \left(t_{k}\right)∥\right) & \frac{\omega^{⊺}\left(t_{k}\right)}{∥\omega \left(t_{k}\right)∥}\sin\left(\frac{T_{\text{s}}}{2}∥\omega \left(t_{k}\right)∥\right) \end{array}\right]}^{⊺}. \end{array}$$

Using this orientation, we transform the measured acceleration into a (slowly drifting) inertial frame, i.e.,


(20) $$\begin{array}{l} {\text{a}}_{\omega }\left(t_{k}\right)≔{\text{q}}_{\omega }\left(t_{k}\right)⊗a\left(t_{k}\right)⊗{\text{q}}_{\omega }{\left(t_{k}\right)}^{-1}. \end{array}$$

In the rotating sensor frame, the gravitational acceleration can point in different directions depending on sensor orientation. In the inertial frame, however, the gravitational acceleration will point in (almost) the same direction regardless of the sensor orientation, and, when integrating, acceleration and deceleration will cancel out. Exploiting this property, we low-pass filter each component of **a**_ω_(*t*_*k*_) by applying a moving average filter with a window length of *T*_*a*_ in forward and reverse direction. Assuming that the change of velocity over the filter window length is small, the resulting filtered acceleration will be dominated by the gravitational acceleration. This filtered acceleration **a**_ω, f_(*t*_*k*_) is then transferred back to the sensor frame


(21) $$\begin{array}{l} a_{\text{f}}\left(t_{k}\right)≔{\text{q}}_{\omega }{\left(t_{k}\right)}^{-1}⊗a_{\omega ,\text{f}}\left(t_{k}\right)⊗{\text{q}}_{\omega }\left(t_{k}\right). \end{array}$$

We then correct the inclination of the gyroscope strapdown integration quaternion **q**_ω_(*t*_*k*_) by using the filtered acceleration as a vertical reference. To this end, we transform the filtered acceleration into the global frame


(22) $$\begin{array}{l} a_{\text{r}}\left(t_{k}\right)≔{\text{q}}_{a}\left(t_{k}\left[-1\right]\right)⊗{\text{q}}_{\omega }\left(t_{k}\right)⊗a_{\text{f}}\left(t_{k}\right)⊗{\left({\text{q}}_{a}\left(t_{k}\left[-1\right]\right)⊗{\text{q}}_{\omega }\left(t_{k}\right)\right)}^{-1}, \end{array}$$

with ${\text{q}}_{a}\left(0\right)≔{\left[\begin{array}{cccc} 1 & 0 & 0 & 0 \end{array}\right]}^{\text{T}}$, and correct the inclination


(23) $$\begin{array}{ll} n\left(t_{k}\right) & ≔a_{\text{r}}\left(t_{k}\right)\times {\left[\begin{array}{ccc} 0 & 0 & 1 \end{array}\right]}^{\text{T}} \end{array}$$

(24) $$\begin{array}{ll} \alpha \left(t_{k}\right) & ≔\arccos\left({\left[\begin{array}{ccc} 0 & 0 & 1 \end{array}\right]}^{\text{T}}\frac{a_{\text{r}}\left(t_{k}\right)}{a_{\text{r}}\left(t_{k}\right)}\right) \end{array}$$

(25) $$\begin{array}{ll} {\text{q}}_{a}\left(t_{k}\right) & ≔{\text{q}}_{a}\left(t_{k}\left[-1\right]\right)⊗{\left[\begin{array}{cc} \cos\left(\frac{\alpha \left(t_{k}\right)}{2}\right) & \frac{n\left(t_{k}\right)}{n\left(t_{k}\right)}\sin\left(\frac{\alpha \left(t_{k}\right)}{2}\right) \end{array}\right]}^{⊺}. \end{array}$$

Multiplication of the gyroscope strapdown integration quaternion and the accelerometer correction quaternion yields the sensor orientation,


(26) $$\begin{array}{l} \text{q}\left(t_{k}\right)≔{\text{q}}_{a}\left(t_{k}\right)⊗{\text{q}}_{\omega }\left(t_{k}\right). \end{array}$$

### 3.9. Foot Velocity and Position Tracking

Using the estimated orientation, we perform double integration of the measured accelerations to estimate the length of each stride, i.e., the horizontal displacement between two adjacent foot-flat phases.

To integrate accelerations, they are first transformed into the reference frame


(27) $$\begin{array}{l} {\text{a}}_{\varepsilon }\left(t_{k}\right)≔\text{q}\left(t_{k}\right)⊗a\left(t_{k}\right)⊗\text{q}{\left(t_{k}\right)}^{-1}. \end{array}$$

Assuming that the velocity is zero in the middle of the foot-flat phase, i.e., at *t*_rest,*i*_, we integrate those accelerations for each stride which yields a velocity


(28) $$\begin{array}{l} {\text{v}}_{i}\left(t_{k}\right)≔T_{\text{s}}\overset{t_{k}}{\underset{\tau =t_{\text{rest},i}}{\sum }}\left({\text{a}}_{\varepsilon }\left(\tau \right)-{\left[\begin{array}{ccc} 0 & 0 & 9.81 \end{array}\right]}^{\text{T}}\right),\;t_{k}\in \left[t_{\text{rest},i},t_{\text{rest},i+1}\right]. \end{array}$$

Due to measurement errors, mainly accelerometer bias, this velocity is usually not zero at *t*_rest,*i*+1_ even if the foot is perfectly at rest. Therefore, we correct this drift linearly over the time duration of the stride:


(29) $$\begin{array}{l} {\text{v}}_{\text{df},i}\left(t_{k}\right)≔{\text{v}}_{i}\left(t_{k}\right)-\frac{t_{k}-t_{\text{rest},i}}{t_{\text{rest},i+1}-t_{\text{rest},i}}{\text{v}}_{i}\left(t_{\text{rest},i+1}\right). \end{array}$$

**Figure 8:**
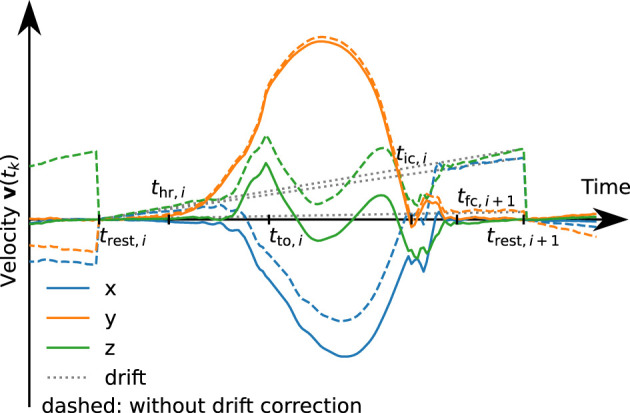
Velocity trajectories with (solid) and without (dashed) linear drift correction. The dotted line represents the subtracted linear drift approximation for stride *i*. For demonstration purposes, the drift has been artificially increased by a factor of 10.

See Figure 8 for an example velocity trajectory with and without drift correction.

By integrating this drift-free velocity over the stride duration, we obtain a position trajectory,


(30) $$\begin{array}{l} {\text{p}}_{i}\left(t_{k}\right)≔T_{\text{s}}\overset{t_{k}}{\underset{\tau =t_{\text{rest},i}}{\sum }}{\text{v}}_{\text{df},i}\left(\tau \right)=:{\left[\begin{array}{ccc} p_{i,x}\left(t\right) & p_{i,y}\left(t\right) & p_{i,z}\left(t\right) \end{array}\right]}^{\text{T}}. \end{array}$$

### 3.10. Stride Length and Walking Speed

We calculate the stride length *L*_*i*_ as the horizontal displacement during the stride *i*. Since **p**_*i*_(*t*_rest,*i*_) = 0,


(31) $$\begin{array}{l} L_{i}≔\sqrt{p_{i,x}{\left(t_{\text{rest},i+1}\right)}^{2}+p_{i,y}{\left(t_{\text{rest},i+1}\right)}^{2}}. \end{array}$$

Note that this method does not make any assumption on the orientation in which the sensor is attached to the foot. Also, note that we integrate from *t*_rest,*i*_ to *t*_rest,*i*+1_ and not from *t*_ic,*i*_ to *t*_ic,*i*+1_ since this makes the zero-velocity assumption more robust.

By dividing the stride length by the stride duration, we obtain the walking speed,


(32) $$\begin{array}{l} v_{i}≔\frac{L_{i}}{T_{\text{stride},i}}. \end{array}$$

### 3.11. Summary of the Estimated Parameters

After performing all steps presented above, the set of proposed methods provides the time instants of the defined gait events, the sensor orientation quaternion for each time instant, and velocity and position trajectories. From those time-based signals, the following gait parameters are extracted for each stride *i*:

- swing duration *T*_swing,rel,*i*_ [%]
- stance duration *T*_stance,rel,*i*_ [%]
- analogously, relative durations for the other gait phases as defined in Figure 4
- stride length *L*_*i*_ [cm]
- walking speed *v*_*i*_ [km/h]
- cadence *c*_*i*_ [steps/min]

Note that all quantities are calculated separately for each stride of each foot. In many cases, only the mean of those values over multiple steps will be of interest. However, this stepwise calculation also allows for analysis of the variance and the detection of trends.

The accuracy of those gait parameters is validated in the next section.

## 4. Experimental Validation

In this section, we aim to show that the less restrictive IMU-based setup combined with the methods proposed in section 3 is able to determine the same parameters as stationary systems that are used in clinical practice while providing similar accuracy. To this end, with a large data set consisting of three different subject groups, we compare the parameters calculated by the proposed methods with values reported by instrumented treadmills.

### 4.1. Setup

**Figure 9:**
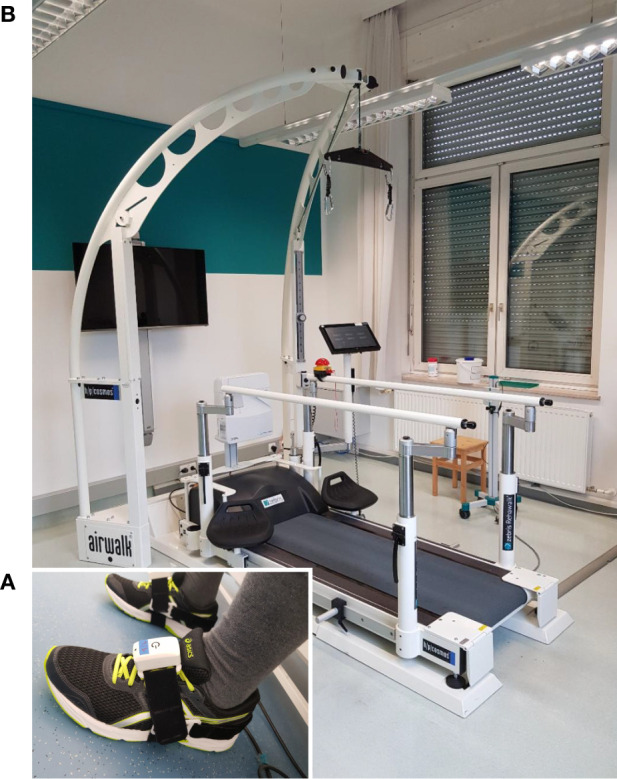
Experimental setup. **(A)** Patient with inertial sensors attached to the shoe. **(B)** Instrumented treadmill at NTK Kapfenberg. Gait parameters are derived from the measurement data of the inertial sensors with the proposed methods and validated against parameters obtained from the instrumented treadmill serving as ground truth.

One PABLO® Lower Extremity inertial sensor (Tyromotion GmbH, Graz, Austria) was attached to each shoe (cf. Figure 9A). The sensors measure angular rate and acceleration at a sampling frequency of 110 Hz. Each sensor has a size of 56 × 34 × 21 mm and transmits the data wirelessly using Bluetooth. The sensors were attached to the subjects' shoes with special Velcro straps.

Zebris Rehawalk instrumented treadmills (Zebris Medical, Isny, Germany) were used as reference systems. Since the data collection took place in various institutions (FH Joanneum Graz, NTK Kapfenberg, Rehabilitation Center Kitzbühel), different systems with identical function were used. See Figure 9B for a picture of the setup at NTK Kapfenberg.

- FH Joanneum (Graz, Austria)
  – Treadmill: h-p-c Mercury Med Treadmill (HP Cosmos, Nussdorf, Germany), walking speed: 0–22 km/h in 0.1 km/h steps, walking surface: 150 × 50 cm
  – Pressure measuring platform: FDM-THM-M-3i (Zebris Medical, Isny, Germany), 120 Hz, sensor area: 108.4 × 47.4 cm, 7,168 sensors.
- NTK (Kapfenberg, Austria)
  – Treadmill: h-p-c Locomotion Med Treadmill (HP Cosmos, Nussdorf, Germany), walking speed: 0–10 km/h in 0.1 km/h steps, walking surface: 150 × 50 cm
  – Pressure measuring platform: FDM-THM-M-2i (Zebris Medical, Isny, Germany), 120 Hz, sensor area: 111.8 × 49.5 cm, 3,432 sensors.
- Rehabilitation Center Kitzbühel (Kitzbühel, Austria)
  – Treadmill: h-p-c Mercury Med Treadmill (HP Cosmos, Nussdorf, Germany), walking speed: 0–22 km/h in 0.1 km/h steps, walking surface: 150 × 50 cm
  – Pressure measuring platform: FDM-THM-M-2i (Zebris Medical, Isny, Germany), 120 Hz, sensor area: 111.8 × 49.5 cm, 3,432 sensors.

### 4.2. Subjects and Experimental Procedure

The data collection was carried out in three different institutions with different groups of subjects. Approval from the ethics committee of the University of Graz was obtained, and an informed consent form was signed by all participants.

*Healthy participants* were recorded at three different walking speeds, each for two minutes: 1.5, 3, and 5 km/h. A prerequisite for participation was the ability to walk on a treadmill at different speeds. The healthy participants (*n* = 39) were recruited from the students at the Physiotherapy Institute of FH Joanneum Graz.

*Non-healthy participants* with affected ability to walk were asked to walk on a treadmill at a self-selected comfortable walking speed. Patients who were unable to walk on a treadmill were excluded during participant selection. The following set of participants were recruited:

- Participants with different neurological diseases (*n* = 36) were recruited from patients who were in neurological inpatient rehabilitation at NTK Kapfenberg at the time of data collection. This comprises 20 post-stroke patients, 6 patients with Parkinson's disease, two with multiple sclerosis, two with meningioma, two after polytrauma, and one patient each with epilepsy, spinocerebellar ataxia, low back pain, and polyneuropathy.
- Participants with various orthopedic diseases (*n* = 62) were recruited from the patients who were in orthopedic inpatient rehabilitation at Rehazentrum Kitzbühel at the time of data collection. Of these, four patients had pathologies in the area of the ankle or lower leg (e.g., ankle joint fractures, tibia fractures), 21 patients at the knee (e.g., osteoarthritis, total knee arthroplasty), 18 patients in the area of the thigh and hip (e.g., osteoarthritis, total hip arthroplasty, femur fractures), 16 patients in the area of the lumbar spine (low back pain, lumbar vertebrae fractures) as well as three patients in whom different body areas were affected (polytrauma, polymyositis).

All participants had time to get used to walking on the treadmill prior to the data collection. All participants were free to use the treadmill support (handrail, fall protection system). For the data collection, two minutes of walking was recorded simultaneously by both systems. IMU data was recorded with a tool of the TyroS software (Tyromotion, Graz, Austria) that allows the export of raw gyroscope and accelerometer data. Zebris data was recorded, analyzed, and exported with the software FDM v1.18.38 (Zebris Medical, Isny, Germany).

### 4.3. Data Processing

For each trial, we obtain the following gait parameters from the Zebris Rehawalk instrumented treadmill:

- loading response duration
- single limb support duration
- pre-swing duration
- swing duration
- stride length
- walking speed
- cadence.

These parameters are reported as averages over the whole trial. The gait phase durations are relative to the stride duration and reported separately for the left and right foot. We add the loading response, single limb support, and pre-swing durations to obtain the stance duration (cf. Figure 3A).

From phases in which the treadmill is not moving and the foot is resting on the ground for approximately 5 s at the beginning and end of each trial, gyroscope turn-on bias is automatically estimated and removed. Using the methods described in section 3, each recorded trial is processed with the parameter values given in Table 2. Note that we use the same set of parameters for all different subject groups and walking speeds in order to demonstrate that the method works well without adjusting the parameters for the specific gait velocity and style.

The sensor attachment used for recording the data sets, as shown in Figure 9A, ensures that one sensor axis is always roughly aligned with the mediolateral axis of the foot. To show that the proposed methods do not make assumptions regarding the sensor orientation, we simulate a random sensor attachment by multiplying all gyroscope and accelerometer measurements with a random rotation matrix that is different for each trial.

Finally, we calculate the same gait parameters as reported by the reference system by averaging the respective parameters, excluding the first and last three strides of each foot, and compare the resulting values to the values reported by the Zebris system. The results are found in the following section.

### 4.4. Results

**Figure 10:**
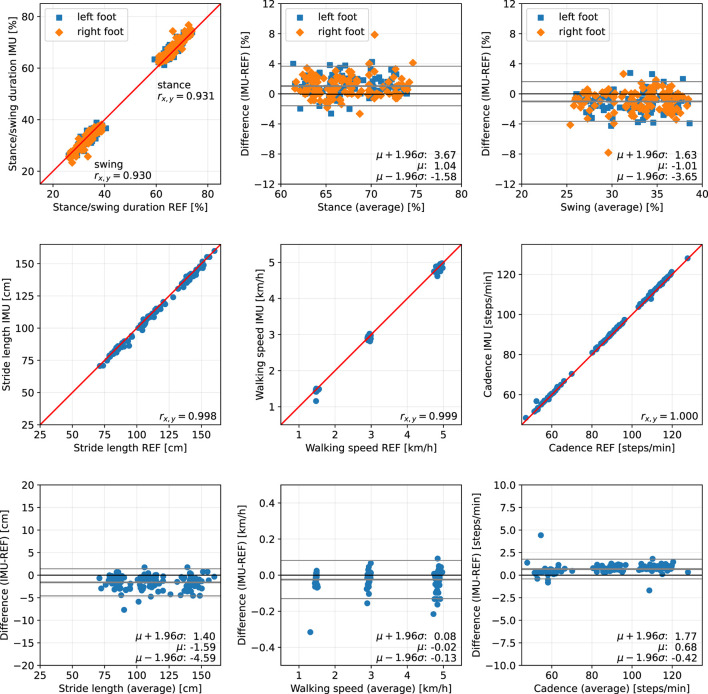
Scatter plots and Bland-Altman plots for stance and swing duration, stride length, walking speed, and cadence of 39 healthy subjects walking at 1.5, 3, and 5 km/h. Red: 45-degree lines (*y* = *x*). Values obtained with the proposed IMU-based methods (IMU) are compared to the ground truth from the Zebris reference system (REF). The average deviation is ~1 % for gait phase durations, below 2 cm for the stride length, below 0.05 km/h for the walking speed, and below 1 step/min for the cadence.

**Figure 11:**
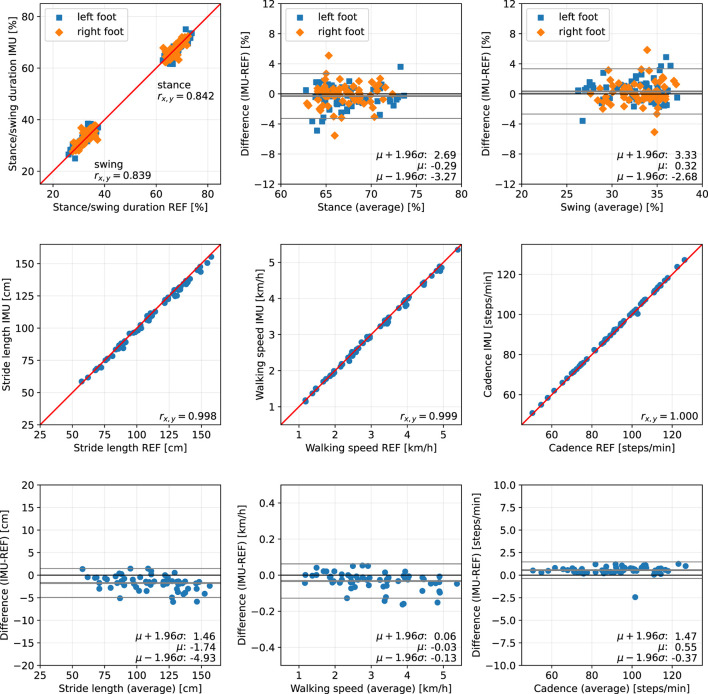
Scatter plots and Bland-Altman plots for stance and swing duration, stride length, walking speed, and cadence of 62 orthopedic patients. Red: 45-degree lines (*y* = *x*). Values obtained with the proposed IMU-based methods (IMU) are compared to the ground truth from the Zebris reference system (REF). The average deviation is below 1 % for gait phase durations, below 2 cm for the stride length, below 0.05 km/h for the walking speed, and below 1 step/min for the cadence.

**Figure 12:**
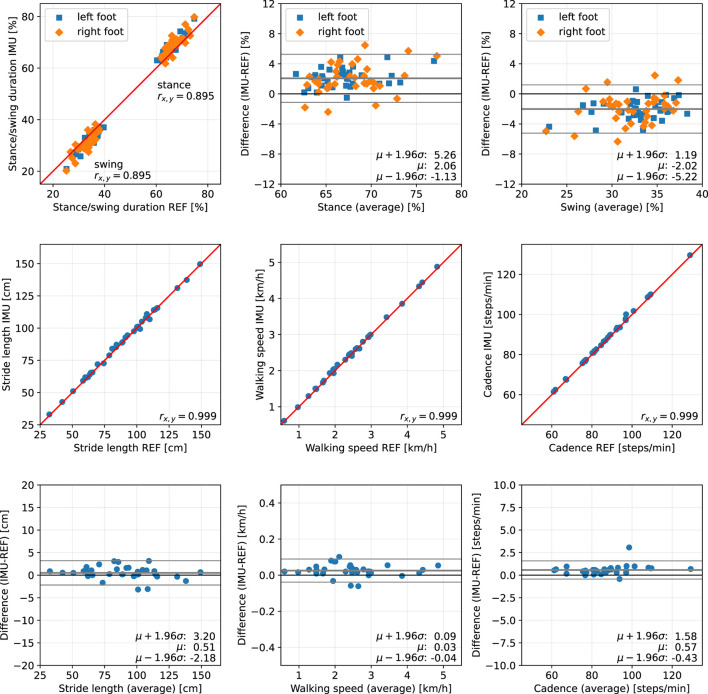
Scatter plots and Bland-Altman plots for stance and swing duration, stride length, walking speed, and cadence of 36 neurological patients. Red: 45-degree lines (*y* = *x*). Values obtained with the proposed IMU-based methods (IMU) are compared to the ground truth from the Zebris reference system (REF). The average deviation is ~2 % for gait phase durations, below 1 cm for the stride length, below 0.05 km/h for the walking speed, and below 1 step/min for the cadence.

For each trial, we first consider the five main parameters stance duration, swing duration, stride length, walking speed, and cadence, and evaluate the difference between the proposed methods (IMU) and the Zebris Rehawalk reference system (REF). The results are presented separately for each of the three subject groups in scatter plots and Bland-Altman plots (50) and can be found in Figure 10, for the healthy participants walking at three different speeds; in Figure 11, for the participants with orthopedic diseases; and in Figure 12, for the participants with neurological diseases.

The error (mean ± standard deviation) for the relative stance duration is 1.04±1.34 % for healthy subjects, −0.29±1.52 % for orthopedic patients, and 2.06±1.63 % for neurological patients. For relative swing duration, the errors are −1.01±1.35 % for healthy subjects, 0.32±1.54 % for orthopedic patients, and −2.02±1.64 % for neurological patients. This means that the average swing/stance duration error is in the range of 1–2 % for all subject groups.

For the stride length, the errors are −1.59 ± 1.53, −1.74 ± 1.63, and 0.51 ± 1.37 cm for healthy subjects, orthopedic patients, and neurological patients, respectively. This means that the average stride length error is below 2 cm for all subject groups.

The mean errors and standard deviations for the walking speed are −0.02±0.05 km/h for healthy subjects, −0.03±0.05 km/h for orthopedic patients, and 0.03±0.03 km/h for means that the average walking speed error is below 0.05 km/h for all subject groups.

The cadence estimates show deviations of 0.68±0.56 steps/min for healthy subjects, 0.55±0.47 steps/min for orthopedic patients, and 0.57±0.51 steps/min for neurological patients. This means that the average cadence error is below 1 step/min for all subject groups.

As an additional evaluation metric, we calculate the mean of the absolute difference (MAD) between the values reported by Zebris and the IMU-based analysis over all trials. Table 3 summarizes the results for the three subject groups and all 215 evaluated trials.

**Table 3:** Deviation between IMU-based and Zebris gait parameters.

	**Stance [%]**				**Swing [%]**	**Stride**	**Walking**	**Cadence**
		**LR [%]**	**SLS [%]**	**PS [%]**		**length [cm]**	**speed [km/h]**	**[steps/min]**
**Healthy subjects (*n* = 39)**								
**MAD**	**1.32**	**1.29**	**1.28**	**1.32**	**1.31**	**1.73**	**0.04**	**0.74**
μ±σ	1.04 ± 1.34	0.97 ± 1.34	−0.96 ± 1.33	1.03 ± 1.34	−1.01 ± 1.35	−1.59 ± 1.53	−0.02 ± 0.05	0.68 ± 0.56
*r*_*x,y*_	0.93	0.93	0.93	0.93	0.93	>0.99	>0.99	>0.99
LoA	−1.58 to 3.67	−1.65 to 3.59	−3.56 to 1.65	−1.60 to 3.66	−3.65 to 1.63	−4.59 to 1.40	−0.13 to 0.08	−0.42 to 1.77
SDC	5.25	5.24	5.21	5.26	5.28	6.00	0.21	2.19
**Orthopedic patients (*n* = 62)**								
**MAD**	**1.14**	**1.12**	**1.14**	**1.07**	**1.16**	**1.94**	**0.04**	**0.63**
μ±σ	−0.29 ± 1.52	−0.33 ± 1.49	0.35 ± 1.49	−0.31 ± 1.44	0.32 ± 1.54	−1.74 ± 1.63	−0.03 ± 0.05	0.55 ± 0.47
*r*_*x,y*_	0.84	0.84	0.85	0.85	0.83	>0.99	>0.99	>0.99
LoA	−3.27 to 2.69	−3.26 to 2.60	−2.57 to 3.26	−3.13 to 2.51	−2.68 to 3.33	−4.93 to 1.46	−0.13 to 0.06	−0.37 to 1.47
SDC	5.96	5.86	5.84	5.65	6.02	6.39	0.19	1.84
**Neurological patients (*n* = 36)**								
**MAD**	**2.26**	**2.20**	**2.23**	**2.21**	**2.22**	**1.09**	**0.03**	**0.60**
μ±σ	2.06 ± 1.63	2.04 ± 1.65	−2.04 ± 1.64	2.06 ± 1.65	−2.02 ± 1.64	0.51 ± 1.37	0.03 ± 0.03	0.57 ± 0.51
*r*_*x,y*_	0.89	0.89	0.89	0.89	0.89	>0.99	>0.99	>0.99
LoA	−1.13 to 5.26	−1.19 to 5.28	−5.26 to 1.18	−1.16 to 5.29	−5.22 to 1.19	−2.18 to 3.20	−0.04 to 0.09	−0.43 to 1.58
SDC	6.39	6.47	6.44	6.46	6.41	5.38	0.13	2.01
**All trials (215 trials)**								
**MAD**	**1.43**	**1.39**	**1.40**	**1.40**	**1.41**	**1.68**	**0.04**	**0.68**
μ±σ	0.83 ± 1.65	0.78 ± 1.65	−0.76 ± 1.64	0.82 ± 1.64	−0.79 ± 1.66	−1.28 ± 1.73	−0.02 ± 0.05	0.62 ± 0.53
*r*_*x,y*_	0.87	0.87	0.88	0.88	0.87	>0.99	>0.99	>0.99
LoA	−2.41 to 4.07	−2.45 to 4.0	−3.98 to 2.46	−2.39 to 4.03	−4.05 to 2.46	−4.68 to 2.11	−0.12 to 0.09	−0.41 to 1.66
SDC	6.48	6.46	6.44	6.42	6.50	6.79	0.21	2.08

*LR, loading response; SLS, single limb support; PS, pre-swing*.

*MAD, mean absolute difference between IMU-based and Zebris values*.

*μ±σ, mean and standard deviation of difference between IMU-based and Zebris values*.

*r_x,y_: Pearson correlation coefficient (p <0.01 for all values)*.

*LoA, limits of agreement, μ−1.96σ to μ+1.96σ*.

*SDC, smallest detectable change, range between both LoA*.

The MAD of the stance and swing durations are ~1.3 % for healthy subjects and orthopedic patients and 2.2 % for neurological patients. Note that we also evaluated the differences for the three sub-phases of stance that the Zebris Rehawalk reference system reports, i.e., loading response, single limb support, and pre-swing. Table 3 shows that we can estimate the duration of those phases with the same accuracy as stance and swing.

To summarize, for all subject groups, the MAD is in the range of 1–2 % for gait phase durations, below 2 cm for the stride length, below 0.05 km/h for the walking speed, and below 1 step/min for the cadence.

## 5. Discussion

In the present contribution, we have proposed a set of methods for spatiotemporal gait analysis based on two inertial sensors attached to the feet. Our methods allow for the calculation of the main spatiotemporal gait parameters that are also reported by stationary laboratory systems: gait phase durations, stride length, walking speed, and cadence. Using a large data set consisting of healthy subjects walking at three different speeds, subjects with orthopedic diseases, and subjects with neurological diseases, we have validated the calculation of those parameters, using a Zebris Rehawalk instrumented treadmill as reference. All parameters show a very strong correlation (Pearson's *r* between 0.83 and 0.99, *p* < 0.01) (51). Figures 10–12 display consistent results over this large and diverse group of subjects. Averaged over all trials, the MAD with respect to the reference system is 1.4 % for the gait phase durations, 1.7 cm for the stride length, 0.04 km/h for the walking speed, and 0.7 steps/min for the cadence.

In clinical practice and research, the presented parameters are used to quantify gait abnormalities and to document changes in the walking behavior of patients. Associations between spatiotemporal gait parameters and functional capacity, or increased mortality, have been demonstrated (52–54). A positive correlation with cardiovascular-related mortality was found for cadence (55). A reduction in walking speed has been shown to correlate with fall risk, frequency of hospitalization, and mortality (56–58). Stride length describes a strong correlation with walking speed according to the research of (59). Slower walking speed, altered gait phase duration, and increased variability of walking increase the risk of falls (60). Furthermore, it was found that psychological modalities, such as fear of falling, can also influence stride length and gait phase duration (61). The minimal clinically important difference (MCID) can be used to determine how precisely these changes must be detected in order to make a statement about their relevance. Despite thorough research, specific values for the MCID could only be found for the walking speed, ranging from 0.36 to 0.72 km/h (62–64). For IMU-based measurement with the proposed methods, the smallest detectable change (SDC) for walking speed is 0.21 km/h and clearly within the MCID for all examined groups. For the other parameters, no reported MCID values could be found, which is consistent with the statement of (29).

The SDC for the cadence is 2.01 steps/min across all studied groups of subjects. This allows for much more accurate changes to be detected than those described as relevant in the literature [e.g., reduction in cadence of 10 steps per minute increases mortality by 4 % (65)]. The achieved SDC for stride length of 5.3 cm in the patients with neurological diseases seems to be sufficiently accurate to capture the differences occurring, for example, in Parkinson's disease (66). The stance and swing phase durations show an SDC of 6.5 % across all trials.

Unlike many existing contributions, we showed that the proposed methods reliably work on patients in addition to healthy subjects and still produce accurate results. This is noteworthy since it has been shown that pathological walking deteriorates the accuracy of many gait analysis methods (45) and specifically the neurologically induced gait abnormalities are challenging for IMU-based gait analysis (29).

A fundamental challenge of IMU-based gait event detection is that IMUs do not directly measure the gait parameters of interest. For toe-off detection, the time instant of load relief cannot directly be measured, and instead, the inversion of the direction of rotation is used. Similarly, initial contact is not detected based on the onset of load but based on the change of acceleration. It is therefore important to properly validate the IMU-based methods by comparing the estimated gait parameters to a reliable ground truth.

As reference system, treadmills instrumented with Zebris pressure measurement platforms were used, which are frequently employed for gait analysis in clinical practice as well as scientific data collection (12). This system shows good reliability (67), but no studies could be found in which the validity of the gait parameters was investigated. It should be noted that due to the length of the pressure sensors (FDM-THM-M-3i: 0.85 cm; FDM-THM-M-2i: 1.27 cm) there may be inaccuracies in the recording of spatial parameters, which may have an effect on the results of the comparative measurements. Moreover, calibration and proper thresholding pose challenges in gait event detection based on pressure measurements (12).

For the neurological patients, the reported duration of stance is, on average, 2 % longer than the reference duration. While this is still a small deviation, it is worth noting because this bias suggests a pattern that is common to this subject group. One likely explanation is that toe-off is being detected later than with the Zebris system. This might be due to a comparatively long phase of load relief that causes the pressure to fall below the threshold too early. Furthermore, the reversal of rotation direction might happen later than for healthy subjects or orthopedic patients. Still, even though both systems measure inherently different phenomena, the observation deviation is only 2 %.

As a replacement for traditional stationary gait analysis systems, which are commonly used in clinical practice, IMU-based gait analysis offers several advantages. Measurement is possible both on treadmills and overground and not restricted to a dedicated laboratory. The small and lightweight IMUs do not restrict the movement of the subject and can be used in conjunction with walking aids such as wheeled walkers. Furthermore, only a very short setup time is required before starting the actual measurement.

Unlike most existing methods (cf. section 2), the proposed method makes gait analysis easier and faster by not requiring any specific sensor attachment, which we demonstrated by simulating a different random sensor-to-foot orientation in each trial. It does not make use of magnetometers and can therefore be used in both indoor and outdoor environments.

While evaluation was limited to the gait phases reported by the reference system, our proposed set of methods further allows for the calculation of many gait phases (Figure 4), i.e., swing and stance for each foot, four unilateral gait phases for each foot, five bilateral gait phases following standard literature (44) for each foot, and finally the distinction between double and single support. To the best of our knowledge, no existing work on IMU-based gait analysis describes the calculation of this set of gait phases.

Besides the more fine-grained gait phases, there are many more parameters that can be extracted, e.g., from the velocity and position trajectories, such as the maximum velocity during swing, foot clearance, and symmetry parameters. While it is not surprising that the prevalence of pressure-based systems has led researchers to focus on features based on ground contact, it is to be expected that the focus of clinical gait analysis will be directed toward other parameters as IMU-based systems become more popular.

Furthermore, miniaturized lightweight sensors with a long battery life open up possibilities for objective gait analysis outside of clinical laboratories. Daily-life gait assessment over the course of multiple days can bring insights that are not possible with short sessions in a laboratory. If patients place the sensors on or in the shoes themselves in an unsupervised telemedicine setting, not requiring the sensor to be oriented in a special way becomes even more important.

Technological advancement also facilitates real-time biofeedback applications. While there are methods for real-time applications that require event detection during a step (41), e.g., to trigger FES, the proposed set of methods is real-time capable in the sense that during walking, sections of data containing a small number of strides can be processed and used to provide feedback to the subject.

The presented work exhibits a few remaining limitations. In the statistical analysis, the gait parameters were averaged over the duration of the trial before comparison with the reference. While it allows for single-stride errors to cancel out, this methodology corresponds well with the use case of clinical gait analysis, in which a subject is asked to walk for several steps, and averaged parameters are then used to assess the gait. An additional stride-by-stride comparison was not performed because the employed reference system can only export averaged gait parameters. In addition, it should be noted that all recordings were made on treadmills and not while walking overground, which has an influence on the movement pattern of gait (68, 69). Despite the known differences between treadmill walking and overground walking, treadmill gait analysis is considered a standard method in clinical practice (70, 71), especially when weight support and handrails are required for safety reasons.

## 6. Conclusion

In the present contribution, we have proposed a set of methods for IMU-based gait analysis. Based on gyroscope and accelerometer measurements from two inertial sensors on the feet, we estimate durations of five gait phases, stride length, walking speed, and cadence. Using a Zebris Rehawalk instrumented treadmill as reference, we validated the proposed methods based on a large data set consisting of healthy subjects (*n* = 39) walking at three different speeds, subjects with orthopedic diseases (*n* = 62), and subjects with neurological diseases (*n* = 36). Averaged over all trials, the MAD with respect to the reference system are 1.4 % for the gait phase durations, 1.7 cm for the stride length, 0.04 km/h for the walking speed, and 0.7 steps/min for the cadence. We also demonstrated that the proposed methods work reliably not only in healthy subjects but also in patients and still provide accurate results under different pathological gait patterns.

This shows that the proposed setup in combination with the proposed methods can accurately calculate relevant gait parameters from the inertial sensor data and thus has the potential to replace traditional stationary gait analysis systems.

Furthermore, we validated that the proposed methods work well regardless of the orientation in which the sensor is attached to the foot, and dedicated calibration movements and magnetometer measurements are completely avoided. The combination of these advantages facilitates long-term ambulatory gait analysis in day-to-day situations without the need for supervision by health professionals.

Future research will focus on the estimation of additional gait parameters, on the validation on stairs and slopes, and the validation against marker-based optical motion capture systems.

## Data Availability Statement

The datasets presented in this article are not readily available because sharing of the data is not covered by the ethical approval. Requests to access the datasets should be directed to Andreas J. Jocham, andreas.jocham@fh-joanneum.at.

## Ethics Statement

The studies involving human participants were reviewed and approved by the Ethics Committee of the University of Graz (GZ. 39/55/63 ex 2017/18, 28 May 2018). The patients/participants provided their written informed consent to participate in this study.

## Author Contributions

DL and TS devised and developed the mathematical method. DL implemented the method, performed the data analysis, and drafted the manuscript. AJ, BG, KA, and MF planned and organized the data collection. AJ, BG, and KA conducted the data collection and post-processed the data. DL, AJ, BG, KA, MF, and TS revised the manuscript. All authors approved the submitted version.

## Funding

We acknowledge support by the German Research Foundation and the Open Access Publication Fund of TU Berlin.

## Conflict of Interest

The authors declare that the research was conducted in the absence of any commercial or financial relationships that could be construed as a potential conflict of interest.

## Publisher's Note

All claims expressed in this article are solely those of the authors and do not necessarily represent those of their affiliated organizations, or those of the publisher, the editors and the reviewers. Any product that may be evaluated in this article, or claim that may be made by its manufacturer, is not guaranteed or endorsed by the publisher.
